# Novelty of Sphingolipids in the Central Nervous System Physiology and Disease: Focusing on the Sphingolipid Hypothesis of Neuroinflammation and Neurodegeneration

**DOI:** 10.3390/ijms22147353

**Published:** 2021-07-08

**Authors:** Maria Ayub, Hee-Kyung Jin, Jae-sung Bae

**Affiliations:** 1 KNU Alzheimer’s Disease Research Institute, Kyungpook National University, Daegu 41566, Korea; maria_ayub14@yahoo.com (M.A.); hkjin@knu.ac.kr (H.-K.J.); 2 Department of Physiology, School of Medicine, Kyungpook National University, Daegu 41944, Korea; 3 Department of Laboratory Animal Medicine, College of Veterinary Medicine, Kyungpook National University, Daegu 41566, Korea

**Keywords:** sphingolipids, sphingosine kinase 1, COX2, specialized pro-resolving lipid mediators, N-acetyl sphingosine, Alzheimer’s disease

## Abstract

For decades, lipids were confined to the field of structural biology and energetics as they were considered only structural constituents of cellular membranes and efficient sources of energy production. However, with advances in our understanding in lipidomics and improvements in the technological approaches, astounding discoveries have been made in exploring the role of lipids as signaling molecules, termed bioactive lipids. Among these bioactive lipids, sphingolipids have emerged as distinctive mediators of various cellular processes, ranging from cell growth and proliferation to cellular apoptosis, executing immune responses to regulating inflammation. Recent studies have made it clear that sphingolipids, their metabolic intermediates (ceramide, sphingosine-1-phosphate, and N-acetyl sphingosine), and enzyme systems (cyclooxygenases, sphingosine kinases, and sphingomyelinase) harbor diverse yet interconnected signaling pathways in the central nervous system (CNS), orchestrate CNS physiological processes, and participate in a plethora of neuroinflammatory and neurodegenerative disorders. Considering the unequivocal importance of sphingolipids in CNS, we review the recent discoveries detailing the major enzymes involved in sphingolipid metabolism (particularly sphingosine kinase 1), novel metabolic intermediates (N-acetyl sphingosine), and their complex interactions in CNS physiology, disruption of their functionality in neurodegenerative disorders, and therapeutic strategies targeting sphingolipids for improved drug approaches.

## 1. Introduction

The fundamental principle of nonpolar acyl chains of lipids to self-aggregate in water lays the basis for cell membrane formation. Although a single lipid species or a group of similar lipids are sufficient to bring about barrier properties, regulate transmembrane solute trafficking, and maintain cellular morphology, the structural composition of these membranes is far more complex and requires the synergy of multiple lipid classes. With advances in our understanding of membrane biology, the potential contribution of various lipid groups in maintaining the flexibility and integrity of cells emphasizes the adaptation pathways observed by cells during diverse biological challenges. Lipids have long been known for their role in structural biology and bioenergetics, such as participating in membrane formation, maintaining cell morphology, and serving as an energy reservoir in times of need. The multifaceted nature of lipids is not only restricted to providing barrier properties or structural and bioenergetics actions, but they also encompass a whole new area of research in lipidomics known as bioactive lipids [1].

Bioactive lipids are a newly defined class of lipids that are actively involved in the regulation of a variety of cellular events. As the name indicates, these molecular factors are subjected to action at the arrival of a specific stimulus and undergo subsequent transitions to cope up with the insult. The term bioactive lipids has been coined owing to their crucial role in regulating the immune and inflammatory responses, maintaining cellular and tissue homeostasis, and clearing the aftermath of a noxious stimulus. The heterogeneous functions executed by these lipids are matched by the diversity of the groups that are within the bioactive lipids. These species include the following major classes: eicosanoids, phospholipids, sphingolipids, endocannabinoids, and the newly discovered specialized pro-resolving lipid mediators (SPMs) [2].

The classical eicosanoids are well known for their role in modulating the inflammatory response by recruiting immune cells to the site of insult, monitoring cell proliferation, and amplifying inflammation [3]. Phospholipids and sphingolipids have a very diversified nature, i.e., they regulate both cell proliferation and apoptosis, mediate cell stress responses and cellular senescence, promote growth signals, as well as facilitate growth arrest [4,5]. However, endocannabinoids function by binding to their specific receptors (type-1 and type-2 cannabinoid receptors) and serve to maintain the homeostatic environment of the respective tissues [6]. SPMs are a newly discovered class of bioactive lipids that have pro-healing and anti-inflammatory functions [7]. Among these lipid classes, sphingolipids are the most prominent signaling molecules due to their heterogeneous functional relevance and the pathologies that they regulate. In particular, the disruption of sphingolipid signaling leads to various diseases with lethal consequences, such as cancer, organ fibrosis, cardiovascular diseases, autoimmune, and neurodegenerative disorders [5]. Recent findings have illustrated the unequivocal contribution of sphingolipid metabolites, i.e., ceramide, sphingosine-1-phosphate (S1P), and N-acetyl sphingosine (N-AS), and enzymes modulating sphingolipid metabolism, particularly sphingosine kinase 1 (Sphk1), in maintaining the central nervous system (CNS) physiology. The crucial contribution of these enzymes and metabolites is particularly highlighted in the etiology of neuroinflammatory and neurodegenerative disorders such as Alzheimer’s disease (AD), Parkinson’s disease (PD), and many others [8]. This emphasizes the importance of these molecular entities in both health and disease and demonstrates the urgency to investigate the mechanistic pathways that regulate the expression of these lipids along with their functional significance.

In this review, we comprehensively discuss the functional significance of various bioactive lipid classes in both periphery and CNS and shed light on the pathways and enzymes that regulate their expression. We also assess the role of sphingolipids in CNS physiology and related disorders, with a focus on neuroinflammation and neurodegeneration, and, in the end, discuss the translational and therapeutic implications that target sphingolipids.

## 2. Functional Significance of Bioactive Lipids in Health

In addition to being the major components of the cell membrane, bioactive lipids function as signaling molecules and possess diverse functional capabilities. Bioactive lipids mediate a plethora of physiological roles, have a relatively short half-life, and act through G-protein-coupled receptors. As mentioned earlier, the subgroups of bioactive lipids contribute toward heterogeneous functions in the body, are recruited at the time of need, and undergo subsequent transitions to deal with the challenge. These lipids are synthesized from omega-3 polyunsaturated fatty acids (PUFAs) and arachidonic acid (AA) and are acted upon by various enzymes such as cyclooxygenases (COXs), lipoxygenases (LOXs), ceramidases, sphingosine kinases (Sphks), and many others. These enzyme systems, and their subsequent expression and mechanistic pathways followed, are crucial in monitoring these lipid classes and their functional potential [9].

**Eicosanoids** play a role in modulating the inflammatory and immune responses in that they secrete inflammatory cytokines and chemo-attractants, recruit leukocytes, and regulate blood flow to the site of insult. This lipid class is further composed of prostanoids (prostaglandins (PGs), prostacyclins, and thromboxanes), leukotrienes, and lipoxins [10]. Eicosanoids are ubiquitously synthesized by the body, i.e., each cell has the machinery to generate one or two major eicosanoids, which in turn function in an autocrine and paracrine manner to maintain local cellular and tissue homeostasis. They act as dynamic regulators of blood pressure homeostasis as they possess the capacity of both vasodepressors (molecules that cause vasomotor depression leading to a reduction in blood pressure) and vasopressors (group of molecules that contract blood vessels and elevate blood pressure) [11]. They also regulate a wide variety of neurophysiological functions such as body temperature regulation, hormonal release, sleep–wake cycle, pain, and inflammatory responses [12]. Some eicosanoids are secreted by the uterus, which then act on the corpus luteum (a temporary endocrine structure in the ovaries responsible for progesterone secretion), causing uterine contractions, otherwise known as labor [13]. These findings demonstrate the significance of eicosanoids in maintaining body homeostasis and the mechanism through which these lipids regulate the physiology of the body from the CNS to the reproductive organs.

**Phospholipids** are the major components of cell membranes based on their structural configuration as they possess both hydrophilic and hydrophobic ends that automatically arrange themselves into membranes. Phospholipid subgroups include phosphatidylcholine (PC), phosphatidylserine (Ptd-L-Ser or PS), phosphatidylinositol (PI), and many others. These lipid classes provide the cells with barrier properties and structural integrity and also monitor membrane trafficking. They permit cells to be selectively permeable to extracellular substances, thus maintaining the cellular homeostatic environment [14]. In addition to contributing toward structural properties, phospholipids ensure cell survival and growth, play a role in endocytosis, phagocytosis [15], and immune surveillance [16], as well as acting as lipid transporters. The CNS is highly enriched in phospholipids, playing a critical role in synaptic transmission, improving cognition, increasing stress resistance, and exerting antioxidant activity [17,18]. These observations significantly indicate the critical role of phospholipids not only in membrane biology but also in other systems, such as the brain.

**Sphingolipids**, besides providing the physiochemical properties to cell membranes, also modulate crucial signaling processes. Sphingolipid metabolic intermediates, including ceramide, sphingosine, and sphingosine-1-phosphate (S1P), are the most explored bioactive sphingolipids. The intracellular and intercellular expression of sphingolipids is modulated by multifaceted complex enzyme systems, among which ceramidase, Sphks (Sphk1 and Sphk2), and S1P lyase are the major regulators [19]. These molecules exert a variety of functions that comprise the regulation of cellular growth, apoptosis, immune cell responses, inflammation, and cell survival. Ceramide has the potential to regulate cell growth, differentiation, cellular senescence, and apoptosis. However, sphingosine has been demonstrated to act as a repressor of cell growth and participate in cell arrest and apoptosis. S1P has antagonizing effects similar to those of ceramide, such as promoting cell survival, proliferation, and resistance to cell apoptosis as well as modulating vasculogenesis, angiogenesis, and inflammation [20,21]. These findings state the substantial significance of sphingolipids in a wide range of physiological functions in the body and emphasize the diversified nature of their metabolites.

**SPMs** are a relatively newly discovered class of bioactive lipids possessing immunoresolvent and anti-inflammatory properties and comprise lipoxins, resolvins, protectins, and maresins. Like other lipid classes, SPMs are endogenously synthesized from PUFAs in response to inflammation or injury. They primarily function to resolve the inflammatory response by restricting the infiltrating immune cells, inhibiting pro-inflammatory cytokines secretion, promoting efferocytosis, and removing cellular debris [22]. Each member of this class not only has a distinct chemical structure but also exerts peculiar functions. For instance, lipoxins obstruct neutrophil infiltration at the site of inflammation, promote macrophage-regulated efferocytosis, and recruit anti-inflammatory monocytes [23]. Resolvins impede the synthesis of pro-inflammatory cytokines, limit leukocyte trafficking, and clear cell debris [24]. However, protectins have neuroprotective properties as well as anti-inflammatory and pro-apoptotic features [25]. Maresins are specifically released by macrophages and are involved in the macrophage-induced phagocytic activity and resolution of inflammation [26]. These studies support the notion that SPMs, along with other classes of bioactive lipids, are critically important in modulating body homeostasis and maintaining the normal physiology.

### Endogenous Regulators of Bioactive Lipid Synthesis: Enzymes and Pathways

The principal pathways involved in the endogenous synthesis of bioactive lipids consist of complex enzyme systems that regulate the production of these lipids depending on the need and time. Each step is closely monitored by a specific enzyme till the very synthesis of the required lipid entity. The initiation of bioactive lipid synthesis is dependent upon the availability of oxidized PUFAs, the starting substrate, which is then acted upon by several enzymes, including Sphks (Sphk1 and Sphk2), COXs, LOXs, cytochrome P450 isoforms, and peroxidases, to generate a plethora of lipid regulators, such as PGs, thromboxanes, resolvins, protectins, maresins, leukotrienes, sphingolipid metabolites, and many others [27]. The major sites and organelles involved in the synthesis of bioactive lipids include the cytosol, endoplasmic reticulum, Golgi body, and mitochondria; hence, all pathways are interconnected, and any dysregulation in the synthesis of one lipid class can eventually affect the expression of neighboring lipids.

The biosynthesis of eicosanoids is intensively monitored by COXs, LOXs, or cytochrome P450 epoxygenase enzymes, all of which yield different products, such as COX-generated eicosanoids, including PGs, prostacyclin, and thromboxanes; LOX-synthesized eicosanoids are lipoxins and leukotrienes, whereas the cytochrome P450 epoxygenase-monitored pathway results in the synthesis of epoxyeicosatrienoic acids (EETs) [28]. Although these eicosanoids are critically significant, COX-generated prostanoids are of special importance as they are actively involved in both homeostatic and inflammatory processes, and extensive research has been conducted on the therapeutic targeting of COX activity. The production of prostanoids is regulated by the COX family of bifunctional enzymes that possess both COX and peroxidase activities and consist of two distinct isoforms, COX1 and COX2. COX1 is ubiquitously expressed by the majority of cells and is the principal source of homeostatic prostanoid production, i.e., these molecular factors serve to maintain body homeostasis and provide cytoprotection. In contrast, COX2 is particularly expressed during the inflammatory response and is the dominant source of prostanoid formation and sustenance of inflammation [29].

All phospholipid groups are derived from phosphatidic acid (starting substrate) through a variety of endogenous pathways and involve multiple enzymes. PC is synthesized through two pathways, viz., the Kennedy pathway and cytosine diphosphate-CDP–ethanolamine pathway. Both pathways involve various enzymes, such as choline kinase, choline/ethanolamine phosphotransferase (CEPT), and phosphatidylethanolamine N-methyltransferase (PEMT), but utilize different initial substrates to synthesize PC [30]. Similarly, PS and PI are synthesized from their respective initiating molecules through the action of phosphatidylserine synthase 1 (PSS1) [31] and phosphatidylinositol synthase (PIS) [32]. These pathways work independently and are interrelated in such a manner that the biosynthesis of phospholipids is an interconnected mechanism.

The biosynthesis of sphingolipids is a rather complex process and comprises three different pathways, including de novo synthesis by the endoplasmic reticulum, the salvage pathway of lysosomes, and finally the sphingomyelinase pathway of the cell membrane. Each pathway yields the same end product, i.e., ceramide, which is then acted upon by various enzymes to result in ceramide-1-phosphate, glycosphingolipids, or S1P. The most important enzymes involved in the synthesis of sphingolipids and their metabolites are ceramidase, which yields sphingosine from ceramide; Sphks, which acts upon sphingosine to produce S1P; both sphingomyelinase (SMase) and sphingomyelin synthase act on ceramide to synthesize sphingomyelin; and glucosylceramide synthase, which generates glycosphingolipids from ceramide [33].

**Figure 1:**
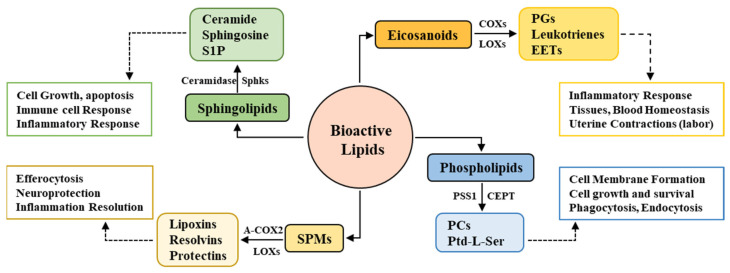
Bioactive lipids, major enzymes, and functional roles.

The action of COXs and LOXs leads to the production of SPMs from AA, eicosapentaenoic acid (EPA), and docosahexaenoic acid (DHA). These enzymes, apart from their contribution in the release of inflammatory eicosanoids, play a prominent role in the synthesis of SPMs. The generation of these SPMs from AA, EPA, and DHA occurs after the catalytic modification of COXs—in particular, COX2. This modification of the catalytic site promotes the anti-inflammatory actions of COX2, i.e., the production of SPMs such as 15-hydroxyeicosatetraenoic acid (15-HETE) from AA, 18-hydroxyeicosapentaenoic acid (18-HEPE) from EPA, and 17-hydroxydocosahexaenoic acid (17-HDHA) from DHA. These anti-inflammatory lipid metabolites then act as substrates for the production of lipoxins, resolvins, and protectins and participate in the resolution of inflammation [34] (Figure 1).

Bioactive lipids consist of heterogeneous subgroups of lipid species, among which eicosanoids, phospholipids, sphingolipids, endocannabinoids, and the newly discovered specialized pro-resolving lipid mediators (SPMs) are the major species. The biosynthesis of each and every single class and species is regulated by specific enzymes, which ensures their time- and need-dependent expression. The major enzymes involved in sphingolipid synthesis are ceramidase, sphingosine kinases, and sphingomyelinase, which yield sphingolipid metabolites, cyclooxygenases, and lipoxygenases, which are responsible for the production of eicosanoid-derived prostaglandins (PGs), leukotrienes, and epoxyeicosatrienoic acids (EETs). Acetylated COX2 (A-COX2) and lipoxygenases yield SPMs consisting of lipoxins, resolvins, and protectins, whereas the synthesis of phospholipid subtypes (phosphatidylcholines (PCs) and phosphatidylserine (Ptd-L-Ser)) is regulated by choline/ethanolamine phosphotransferase (CEPT), phosphatidylserine synthase 1 (PSS1), and many others. These complex biosynthetic pathways and the diverse classification of bioactive lipids are based on the various functions regulated by subsequent classes; for example, sphingolipids regulate cell growth, apoptosis, and inflammatory and immune responses; SPMs are involved in inflammation resolution, whereas eicosanoids act as inflammation amplifiers; and phospholipids primarily regulate cell membrane physiology.

All these enzymes are crucial and closely monitor each and every step of bioactive lipid synthesis. However, Sphks (particularly Sphk1) and COX2 have gained more attention compared to the other enzymes due to their multifunctional enzymatic activities. Sphk1 not only regulates the synthesis of S1P from sphingosine but also modulates the COX2 enzyme activity and therefore the expression of prostanoids and SPMs. Similarly, COX2 not only controls the production of inflammatory eicosanoids but also participates in SPM synthesis and is thus believed to possess anti-inflammatory properties [35]. Sphk1 and COX2 thus act as a bridge connecting the biosynthesis of lipids and their functional contribution, which will be explored in detail in subsequent sections in this review. This contradictory functional characteristic of COX2 and the bridging connection of Sphk1 and COX2 are particularly prominent in the CNS physiology and pathology; therefore, there exists an urgency to explore the functional and pathological contribution of sphingolipids in the CNS.

## 3. Compartmentalization of Sphingolipids in the CNS as Drivers of Physiology and Pathology

Among the various subgroups of bioactive lipids that inhabit the CNS, sphingolipids have gained much attention due to their abundant expression and participation in both maintaining the CNS homeostasis and initiating inflammation or degeneration. Not only the subclasses of sphingolipids but also the cellular processes that they regulate are equally diverse. These molecules regulate the production of pro-inflammatory eicosanoids, control neuronal cell apoptosis, monitor glial cell activity, and maintain CNS immune cell homeostasis. The diversified functions of sphingolipids in the CNS are also explained by the types of cells that express these metabolic intermediates; for example, ceramide has an apoptotic effect on neuronal cells while it shifts the molecular phenotype of astrocytes and microglia to pro-inflammatory and promotes the release of inflammatory factors. However, S1P tends to induce morphological changes in glial cells and participates in the sustenance of neuroinflammation, while providing a protective role in neuronal cells [36]. Besides the importance of CNS sphingolipids in modulating various process, signaling from the periphery, especially the gut (known as the gut–brain axis), has also emerged to monitor the lipid levels in the CNS [37]. This diversified nature of sphingolipid metabolites to mediate heterogeneous physiological functions in the CNS has gained much attention in neuropathologies as well. Dysregulation of these metabolites either at the expression level or signaling level has been linked with neurodegenerative disorders such as AD and PD, as well as some other neurodegenerative diseases. Thus, the following sections highlight the physiological significance of these metabolites in the healthy CNS and how their dysregulation leads to associated neuropathologies.

**Ceramides** are generally considered apoptotic molecules; however, their roles in cellular homeostasis and CNS physiology are far beyond just being pro-apoptotic molecular entities. Neutral sphingomyelinase (nSMase), an enzyme that monitors the levels of ceramide, is expressed in the hippocampus and regulates synaptic transmission by modulating ceramide concentrations. At the synaptic level, nSMase controls the NMDA receptor expression at the postsynaptic excitatory neurons [38]. It has been reported that nSMase-deficient mice have unbalanced sphingolipid levels that are responsible for impaired synaptic plasticity and spatial memory [39]. Although these findings indicate the unequivocal participation of ceramides for maintaining the normal synaptic plasticity and transmission in the CNS, any disruption in ceramide levels or functions is more prominently highlighted in neuropathologies.

Ceramides are particularly associated with the inflammatory profile in peripheral diseases, such as cystic fibrosis, inflammatory bowel disease, and airway inflammation [40], and also in CNS disorders, such as neuroinflammation, multiple sclerosis, and Alzheimer’s disease (AD) [41]. In the brain, ceramides have been reported to cause neuronal cell apoptosis through mitochondrial dysregulation, generation of excessive reactive oxygenspecies, or activation of cell death pathways such as caspase 3/9 [42,43]. Not only do ceramides activate cell death pathways and induce apoptosis of neurons, but they also modulate cellular senescence and inhibit differentiation. However, these molecules shift the glial cell response toward the pro-inflammatory, through the activation of microglial NF-κB signaling and promotethe release of inflammatory molecules such as tumor necrosis factor-α (TNF-α), interleukin-1β (IL-1β), and interleukin-6 (IL-6) by astrocytes, which leads to neuroinflammation [44]. In AD pathology, multiple studies have reported that sphingolipid metabolism is altered, with an overall shift from S1P to a ceramide gradient, which enhances neuronal apoptosis and therefore neurodegeneration [45,46]. In AD, amyloid beta (Aβ) has been observed to act as a positive regulator of SMase activity with an increase in Aβ deposition; SMase activity is enhanced, resulting in abnormally high levels of ceramide in the brain. Neuronal cell death and neuroinflammation are then initiated by these ceramides, which are the hallmarks of AD pathology [47]. These studies altogether highlight the irrefutable importance of ceramides in both synaptic plasticity and neuroinflammation and neurodegeneration and indicate the therapeutic potential of targeting these molecules.

**S1P** plays a significant part in diverse immunological processes such as cell survival, immune cell switching, and cell trafficking and therefore maintains cellular homeostasis. Multiple studies have demonstrated that the CNS has a well-established system of enzymes that are involved in S1P production and expresses S1P receptors (S1PR1–S1PR5) that regulate a variety of CNS physiological functions [48]. The major contributors to S1P expression in the brain are the endothelial cells that form the blood–brain barrier (BBB), and these cells are enriched with S1PR1 receptors. The brain endothelial cell S1PR1 signaling regulates the functions of the BBB tight junction proteins, and any disruption to this signaling pathway will lead to the structural disintegration of BBB and a leaky CNS phenotype [49]. At the synaptic level, S1P regulates the localization of synaptic proteins, including synapsin-I in presynaptic terminals, monitors the glutamate expression and its release from synaptosomes, and therefore plays a critical role in the modulation of synaptic transmission [50,51]. S1P levels in the brain are dominantly regulated by Sphk1 actions, and a dysregulation of this S1P–Sphk1 signaling has been identified in numerous CNS pathologies, particularly in AD [52].

During the inflammatory response, S1P acts to promote the ongoing insult by enhancing the inflammatory signatures of glial cells by altering the morphology of microglia and astrocytes, promoting their proliferation and migration, and increasing the synthesis of pro-inflammatory cytokines [53,54]. LPS-treated mice displayed abnormally elevated levels of Sphk1 activity, which increased the expression of S1P and pro-inflammatory molecules, whereas a reduction in Sphk1 activity restored the morphology of glial cells, impeded their migration, and inhibited their proliferation [52]. This Sphk1–S1P signaling is exacerbated in AD mice models, and an imbalance in S1P levels has also been observed in human brain studies. These data support the inflammatory contribution of glial cells in Sphk1–S1P signaling, whereas neuronal Sphk1 has been reported to demonstrate contradictory functions, i.e., neuronal Sphk1 regulates microglial phagocytic functions and COX2 activity, and decreased expression of this signaling has been observed in the AD pathology [55].

### 3.1. Deregulation of Neuronal Sphk1–S1P Signaling in AD

One of the potential mechanisms that Aβ deposition disrupts during AD pathogenesis is the deregulation of neuronal Sphk1–S1P signaling in the CNS. Cellular studies have reported that a shift in sphingolipid rheostat, i.e., the transition from a cell survival mediator (S1P) to a pro-apoptotic ceramide, occurs during AD pathogenesis [44]. Elevated levels of ceramides have been reported, while a reduction in neuronal S1P levels has been observed along with alterations in Sphk1 enzyme concentrations. The Sphk1-modulated sphingolipid metabolism disruption is severely highlighted in the etiology of AD mice models, and abnormally altered levels of ceramides and S1P are observed in patients with AD [56].

Postmortem studies on patients with AD have reported a reduction in neuronal Sphk1 activity, which consequently monitors S1P levels in the brain. Sphk1 and S1P are affected by Aβ deposition in the brain, where Sphk1–S1P signaling inversely correlates with increasing Aβ concentrations, i.e., the more the Aβ is deposited, the lesser is the Sphk1–S1P activity. Conversely, S1P lyase (the enzyme that regulates the final step of S1P catalytic conversion into end products) is elevated in AD pathology and exhibits a positive correlation with Aβ deposition, i.e., the greater the Aβ pathology, the higher the S1P lyase activity and the lower the S1P levels in the brain [57]. This overall shift in sphingolipid metabolism results in lower levels of S1P in the brain, which is a pro-survival and neuroprotective metabolite. This transition leads to sustained neuroinflammation and exaggerated neuronal loss. In addition, an alteration in Sphk1–S1P activity has been found to be dominant in some CNS regions compared with that in other regions, i.e., decreased S1P expression, reduced Sphk1 activity, and elevated S1P lyase levels were detected in the CA1 region of the hippocampus and gray and white matter of the temporal gyrus in patients with AD; both these regions regulate memory formation and spatial learning in the brain [56]. In vitro studies of reciprocating AD pathology through treatment of neuronal cells with Aβ peptide have also demonstrated similar patterns to those of Sphk1–S1P signaling. Aβ-treated neuronal cells were found to exhibit elevated ceramide concentrations, reduced Sphk1 activity and S1P levels, and enhanced cellular apoptosis. However, overexpression of Sphk1 conferred neuronal cell cytoprotection, restored the sphingolipid balance toward neuroprotective S1P, and reduced cell death [58].

Altogether, these findings provide a potential direction for evaluating AD pathology in view of sphingolipid metabolism, with a particular focus on the protective role of neuronal Sphk1–S1P signaling. Although this Sphk1–S1P signaling has been investigated in AD pathogenesis, recent research has indicated potential new roles of Sphk1 in both CNS physiology and pathology. The latest findings have suggested a new functional characteristic of Sphk1, i.e., Sphk1 acts as an acetyltransferase for COX2 in the brain, and have mentioned the crucial contribution of the Sphk1 and COX2 bridging connection in the CNS.

### 3.2. Sphk1 and COX2 Together Act as a Bridge between CNS Homeostasis and AD Pathology

In the steady-state CNS, a perfectly harmonious coordination of Sphk1 and COX2 is present, which plays a very crucial role in the maintenance of the homeostatic environment, and disruption of this harmony results in neuroinflammation and eventually neurodegeneration. During physiological conditions, Sphk1 is expressed by various brain regions and neuronal cells, and it regulates diverse cellular processes, e.g., neurogenesis, axonal growth, and the release of neurotransmitters [59,60]. Sphk1 in the brain has different signaling pathways, such as ATP-dependent sphingosine metabolism to S1P, Sphk1–S1P signaling, and modulation of COX2 expression by functioning as an acetyltransferase. It has been recently reported that Sphk1 has the ability to catalytically modify COX2 and acetylate it at the serine 565 site. As mentioned earlier, COX2 has both inflammatory and anti-inflammatory activity and, depending on the stimulating cue and the enzymes modulating its activity, can result in either of two characteristics. During the inflammatory response, COX2 generates a large number of prostanoids, which sustains the ongoing insult [61]. Non-steroidal anti-inflammatory drugs (NSAIDs) such as aspirin are the first drug of choice used to treat inflammatory diseases. The major pathway followed by aspirin is acetylation of COX2 and shifting its activity toward the anti-inflammatory domain, which consequently synthesizes the immunoresolvent SPMs [62]. This mechanism is also observed in animal studies, where Sphk1 functions as an endogenous regulator of COX2 acetylation and SPM secretion, rather than exogenously administered drugs.

This triad of Sphk1–COX2–SPMs is severely altered in AD pathogenesis in both animal models and human studies. A recent study showed that neuronal Sphk1 expression was reduced in AD mice compared with that in the control group. The brains of AD mice displayed reduced mRNA expression of neuronal Sphk1, but no considerable difference was observed between the glial cell population, which highlights the cell-dependent activity of Sphk1. Due to reduced Sphk1 expression and activity, the COX2 anti-inflammatory function and the synthesis of SPMs were significantly disturbed. The study also reported that during AD pathology, Sphk1 expression was decreased, shifting the COX2 activity toward prostanoid synthesis and impeding the production of SPMs [35]; in short, this explains the poor resolution of inflammation due to the abrogated communication between Sphk1, COX2, and SPMs. The dysfunction of this trilogy causes a dysregulated crosstalk between neuronal and glial cells, affects the microglial phagocytosis of Aβ, increases the production of pro-inflammatory cytokines such as TNFα, IL-1β, and IL-6, and fails to inhibit the infiltration of peripheral immune cells into the CNS. These abnormalities, along with reduced SPM release, sustain the neuroinflammatory phenotype of the AD brain, increase the Aβ load, and eventually lead to neurodegeneration.

**Figure 2:**
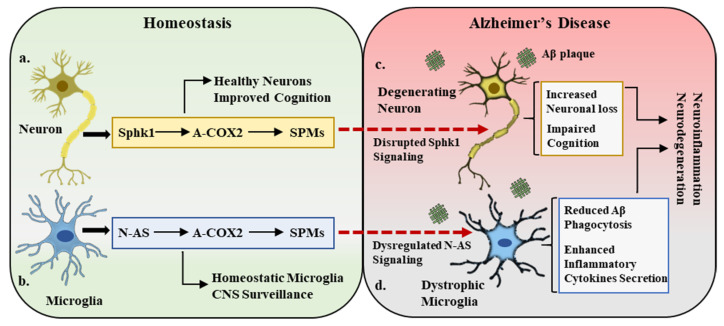
Sphk1–COX2–SPM trilogy in homeostasis and Alzheimer’s disease. (**a**). Under homeostatic conditions, neuronal cells express sphingosine kinase 1 (Sphk1), which is a potent regulator of COX2 acetylation (A-COX2) and subsequent SPM synthesis. This Sphk1–COX2–SPM trilogy maintains the central nervous system (CNS) homeostatic environment, improves cognition, and maintains healthy neurons. (**b**). In a parallel manner, the microglial compartment in the CNS expresses N-AS, which also acetylates COX2 and monitors SPM expression. This N-AS signaling maintains the microglial activity in balance and participates in CNS surveillance. (**c**,**d**). In Alzheimer’s disease pathology, amyloid beta (Aβ) deposition results in the disruption of Sphk1 signaling in neurons and N-AS expression in microglia, leading to neuronal loss (degenerating neurons), defective microglia (dystrophic microglia), cognitive impairment, and overall inflammatory and neurodegenerative environment.

However, elevated levels of neuronal Sphk1 improved the AD pathology by restoring the COX2 anti-inflammatory activity, synthesizing neuronal SPMs, and reestablishing neuronal and glial cell communication. Increased Sphk1 levels also reduced the COX2-triggered prostanoid secretion by modifying its catalytic domain at serine 565. This acetylation promoted the production and release of neuronal SPMs, particularly 15-R-lipoxin A4 (15-R-LxA4), which is a potential resolver of inflammation. These SPMs not only effectively resolved neuroinflammation but also improved the microglia-mediated phagocytosis of Aβ plaques, which caused reduced Aβ burden in the brain. To the best of our knowledge, this is the first study to demonstrate a novel role for Sphk1 as an acetyltransferase for COX2 and describe the complex interactions between Sphk1 and COX2 activity. The study also postulates a bridging connection between Sphk1 and COX2 in maintaining CNS homeostasis and highlights the potential contribution of the Sphk1–COX2–SPM trilogy in AD (Figure 2a,c) [55].

### 3.3. N-acetyl Sphingosine: Direct Modulator of COX2 Acetylation in the CNS

Previous observations demonstrating neuronal Sphk1 acting as an acetyltransferase and regulator of COX2 acetylation and the subsequent synthesis of neuronal SPMs, which then act on microglia and modulate their phagocytic activity, indicate the indirect monitoring of microglial phagocytic activity by neurons. Although this neuronal Sphk1 and the microglial phagocytosis crosstalk are severely altered in AD pathology and subsequently exacerbate the disease, the direct controllers of the microglial phagocytosis of Aβ remain to be investigated. In a recent study by our research group, we reported the direct regulator of microglial phagocytic activity independent of neuronal tampering. The study reported that N-AS, an intermediate product in sphingosine metabolism, acetylates COX2 at serine 565 without neuronal involvement. It was observed that the Sphk1-regulated sphingosine metabolism involves the production of N-AS, wherein acetyl-CoA binds to the ATP-binding domain in Sphk1 and transfers this acetyl group to sphingosine, leading to the production of N-AS.

Under physiological conditions, both neurons and microglia synthesize N-AS, whose expression and function are affected by Aβ deposition in AD. Our study emphasized the importance of microglial-generated N-AS, as its levels were found to be severely reduced compared with those of neuronal N-AS in AD pathology. During AD progression, as Aβ plaques are increased, Sphk1 activity is disturbed, which results in decreased levels of N-AS, whereas microglial N-AS expression is reduced due to the lower availability of acetyl-CoA, an essential component in N-AS synthesis. Previous reports have demonstrated that AD mice models (2576 Tg and 3xTg) have reduced acetyl-CoA concentrations, which affects the microglial mitochondrial activity by inhibiting the pyruvate dehydrogenase complex, which ultimately results in defective microglia [63,64]. These findings are consistent with our microglial N-AS study, which urged us to investigate the role of N-AS in regulating microglial phagocytosis and its ultimate contribution to AD pathology.

It has also been demonstrated that a decrease in the synthesis of N-AS by acetyl-CoA-deficient microglia resulted in lower acetylation of COX2, which subsequently affected the release of SPMs and thus caused poor resolution of neuroinflammation and AD pathology. It has been observed that SPMs such as LXA4 and RvD1 play a significant role in maintaining the phagocytic functions of microglia [65,66]; therefore, N-AS-deficient microglia exhibit not only reduced SPM synthesis but also defective phagocytosis. Restoring the acetyl-CoA expression or exogenous administration of synthetic N-AS did rescue the AD pathology by improving COX2 acetylation, SPM secretion, and microglia-mediated Aβ phagocytosis. N-AS was able to restore the microglia’s multiple functional characteristics and also aid in reducing the gene expression of pro-inflammatory cytokines and limiting the infiltration of peripheral immune cells into the brain. This study reported a novel and direct regulator of COX2 anti-inflammatory actions and microglia functionality and demonstrated the need for further studies to explore the therapeutic potential of N-AS in AD (Figure 2b,d) [67].

### 3.4. Sphingolipids in the Pathobiology of Parkinson’s Disease

Parkinson’s disease (PD) is the second most common neurodegenerative disorder and affects ~1% of the population over 60 years of age [68]. Neuropathological hallmarks of PD include striatal dopamine deficiency due to neuronal loss in substantia nigra, and aggregation of α-synuclein, which results in the formation of lewy bodies. On the other hand, symptomatic hallmarks of PD include motor dysfunctions characterized by tremor, rigidity, slow moments, and impaired balance and gait. The pathophysiology of PD has been investigated from genetic predisposition, environmental factors, and aging aspects. The major mechanistic disruptions observed in PD pathology involve mitochondrial dysfunction, alterations in dopamine metabolism, generation of reactive oxygen species, microgliosis, and neuroinflammation, which altogether leads to neurodegeneration and the onset of motor dysfunctions [69].

In PD studies, sphingolipids have gained a great deal of attention in recent years, both at the genetic and metabolic levels, and will be discussed briefly in this section. Lipidomic analysis in postmortem PD brain tissue has shown an imbalance of ceramide and sphingomyelin levels along with increased levels of ceramides in blood plasma [70,71]. Glucocerebrosidase (GBA), a lysosomal enzyme that metabolizes glucosylceramide into free ceramide and glucose, is one of the top genetic contributors to PD development [72]. GBA mutations result in imbalanced ceramide levels and contribute to early PD development, rapid progression, and severe psychiatric symptoms. GBA is an important enzyme in α-synuclein degradation and is reported to protect against α-synuclein aggregation in the brain [73].

Some studies have reported a negative correlation between the aggregation of α-synuclein in neuronal cells and S1PR1 signaling, thus affecting the vesicular trafficking regulated by S1PR1 [74]. The studies by Joanna B Strosznajder’s group on the association between PD and sphingolipid metabolism have uncovered the potential role of Sphk1 in modulating the disease pathology. Their research reported that in a PD in vitro model of MTPT/MPP+ (1-methyl-4-phenyl-1,2,3,6-tetrahydropyridine (MPTP), 1-methyl-4-phenylpyridinium), Sphk1 activity was significantly reduced, which enhanced α-synuclein secretion and the activation of genes associated with apoptosis. While the administration of PPX (pramipexole), a dopamine D2/D3 receptor agonist commonly used in PD therapy that acts via modulating Sphk1 activity, partially rescued the PD pathology [75,76]. These aforementioned studies altogether point towards the critical participation of different sphingolipid metabolites and enzymes in PD prognosis. Interestingly, sphingolipid studies in PD have shown similarities with AD as ceramide levels were increased, Sphk1 activity was significantly reduced, and Sphk1/S1P signaling was disrupted, as reported in AD as well. Based on the studies conducted on the Sphk1 and N-AS role in AD and the similarities between AD and PD in regard to sphingolipids, they may presents new targets (Sphk1 and N-AS) to explore in the PD pathogenesis.

### 3.5. Sphingolipids and Other Neurodegenerative Disorders

**Huntington’s disease** (HD) is a progressive brain disorder caused by a dominant mutation in the Huntington (HTT) gene. HD is characterized by movement disorder such as random uncontrolled movements and imbalance; behavioral abnormalities like depression, anxiety, and psychosis; and cognitive impairment such as impaired learning and executive functions (problem solving, concentrating, and multitasking) and dementia [77]. Animal models of HD have reported that an imbalance in sphingolipid metabolite levels and enzyme activity occurs in the early stages of the disease’s development [78]. The study of HD postmortem brains and animal models has shown reduced Sphk1 activity in the cortex along with increased sphingosine-1-phsophate lyase 1 (SGPL1; an enzyme involved in the metabolism of S1P) [79]. The R6/2 mice model of HD has shown reduced levels of S1P in the brain, leading to abnormal neuronal activity, increased brain atrophy, motor dysfunction, and lower rates of survival [78]. All of these phenotypic and cellular pathologies were rescued upon FTY720 (S1PR agonist) administration [80].

**Amyotrophic lateral sclerosis** (ALS), also known as motor neuron disease, is a neurodegenerative neuromuscular disorder, characterized by the loss of motor neurons that control movement, muscle wasting, paralysis, and severe deregulation of lipid metabolism [81]. Mouse models of ALS display increased levels of ceramides in the spinal cord, immune cell dysregulation, along with disrupted exosome and lysosome secretion [82,83]. Disturbed ceramide, sphingosine, and sphingomyelin levels are reported to correlate with ALS severity together with Sphk1 activity and expression [83]. Altered levels of sphingomyelin and some other sphingolipids have been observed in the cerebrospinal fluid of ALS patients, pointing towards their clinical relevance. By contrast, hyperlipidemia (high levels of lipids in blood) appears to have a protective effect in ALS, and enhanced survival in an ALS mice model and slow progression of disease in ALS patients have been reported following hyperlipidemic diet consumption [84].

These studies of HD and ALS highlight the genetic and functional contribution of sphingolipid metabolites in the etiology of these disorders and why they should be explored in detail from a therapeutic perspective. Interestingly, HD and ALS have shown similar disturbances in sphingolipid metabolism to PD and AD. The investigation of Sphk1, N-AS, and COX2 acetylation in these disorders can unravel possible mechanisms and pave the way towards the design of relevant therapeutics for neurodegenerative disorders.

## 4. Conclusions

### Therapeutic Implications and Future Directions

The biology of sphingolipids has been extensively explored in the peripheral system, and the associated pathologies have also been thoroughly investigated; however, despite being major structural components of the brain anatomy, the functional significance of sphingolipid signaling in the CNS remains underexplored. The majority of published data emphasize the involvement of sphingolipids in inflammatory disorders, such as asthma, chronic pain, rheumatoid arthritis, and infections [85], and some recent findings indicate their role in neuroinflammation and neurodegeneration as well [36,86]. As mentioned earlier, aspirin is the treatment of choice for inflammatory illnesses, which acts as a nonselective inhibitor of COX2 enzymatic activity and resolves inflammation by abrogating the synthesis of prostanoids. This rescue effect of aspirin has also been reported to reduce AD pathology in animal disease models; however, in human studies, the effects have been contradictory, which indicates a limitation of these drugs and demonstrates why endogenous regulators must be explored to develop specific drug targets [87].

The above-described findings highlight two newly discovered endogenous modulators, viz., Sphk1 and N-AS, that maintain CNS homeostasis, and any disruption in their signaling can result in pathological states. One study highlights the indirect monitoring of microglia by neuronal Sphk1, which acts as an acetyltransferase for the COX2 enzyme and acetylates it at serine 565, which provides its anti-inflammatory functions. The resulting acetylated COX2 aids in the synthesis of SPMs by neurons, which subsequently act on microglia, enhancing their Aβ phagocytosis. Although Sphk1 is a potential candidate in AD pathology, it is an indirect regulator of microglial phagocytic activity that involves neuronal actions, whereas N-AS, a sphingosine metabolic intermediate, is a newly reported direct regulator of COX2 acetylation and microglial phagocytic activity. Overall, these studies describe the potential of sphingolipid expression and their enzyme systems in regulating the CNS homeostatic state and lay the foundation for further exploration of the therapeutic potential of these mediators. Sphk1 and N-AS as drivers of COX2 acetylation have been well explored in AD pathology, as discussed in the above findings, but they remain to be investigated in other neurodegenerative disorders such as PD, HD, and ALS. The reported literature has highlighted an interesting point of similar changes in sphingolipids observed among AD, PD, HD, and ALS, and this opens new avenues to explore the Sphk1, COX2, and SPM trilogy, and N-AS signaling in other neurodegenerative disease as well.

One of the challenges faced by scientists in the neuroscience community is the development of therapeutics that have the potential to cross the brain barriers and enter the CNS to effectively target the site of insult. Neuronal Sphk1 presents a potential pathway that can be targeted in AD pathogenesis, and N-AS indicates its therapeutic potential, i.e., to replicate it exogenously as a therapeutic for AD. N-AS is an acetylated lipid itself and has the capacity to cross the BBB when administered, which again supports its therapeutic relevance. Moreover, accumulating evidence has suggested that modifications of lifestyle, particularly the dietary intake of certain bioactive foods, can influence the development of neuroinflammation through a multitude of mechanisms such as modulating microbiota composition, affecting neurotransmitter secretion, and protecting against oxidative stress. Bioactive compounds such as omega-3 fatty acids, fat-soluble vitamins, isothiocyanates, and carotenoids seem to have rescue potential in AD pathology, i.e., a reduction in Aβ deposition, providing antioxidant activity and anti-inflammatory activity and overall protection against cellular apoptosis [88]. The beneficial effects of consuming bioactive foods and maintaining a proper lifestyle are not limited to AD only but are also reported in PD and other neurodegenerative disorders [89]. Further investigations of these molecules (endogenous regulators and dietary compounds) and pathways will not only provide a translational aid but will also allow the discovery of novel lipid mediators involved in CNS physiology and pathology.
